# Can the parkland grading scale predict the difficulty of laparoscopic cholecystectomy? A new approach to validation

**DOI:** 10.1186/s12893-023-02036-0

**Published:** 2023-05-25

**Authors:** Ya-qi Liu, Chao Wang, Xuan Cai, Zhi-xue Zheng, Jing-tao Bi

**Affiliations:** 1 Department of General Surgery, Beijing Jishuitan Hospital, Beijing, 100035 China; 2 Beijing Research Institute of Traumatology and Orthopaedics, Beijing Jishuitan Hospital, Beijing, 100035 China

**Keywords:** Parkland Grading Scale, Laparoscopy, Laparoscopic cholecystectomy, Cholelithiasis

## Abstract

**Background:**

The Parkland Grading Scale (PGS) is an intraoperative grading scale to stratify gallbladder disease severity during laparoscopic cholecystectomy (LC). We evaluated the usefulness of the PGS in predicting the difficulty levels of LC procedures using a novel approach.

**Methods:**

A total of 261 patients diagnosed with cholelithiasis and cholecystitis who underwent LC were assessed. The PGS and the surgical difficulty grading system were used to evaluate surgical procedures by reviewing the operation videos. Clinical baseline characteristics and post-treatment outcomes were also recorded. Differences between the five PGS grades in terms of surgical difficulty scores were analyzed using the Jonckheere-Terpstra test. The relationship between PGS grades and surgical difficulty scores was assessed using Spearman’s Rank correlation. Finally, the linear trends between morbidity scores and PGS grades were evaluated using the Mantel-Haenszel test.

**Results:**

There was a significant difference in the surgical difficulty scores for the five PGS grades (*p* < 0.001). In pairwise comparison, each grade (1–5) was significantly different from the others (*p* < 0.05) in terms of surgical difficulty, except Grade 2 vs. 3 (*p* = 0.07) and Grade 3 vs. 4 (*p* = 0.08). There was a significant correlation between PGS grades and surgical difficulty scores (*r*_*s*_ = 0.681, *p* < 0.001). There was also a significant linear association between morbidity and PGS grades (*p* < 0.001). Spearman’s R value was 0.176 (*p* = 0.004).

**Conclusion:**

The PGS can accurately assess the surgical difficulty level of LC. The precision and conciseness of the PGS make it suitable for use in future research.

## Background

Laparoscopic cholecystectomy (LC) is widely performed by general surgeons for cholelithiasis [1]. Differences in gallbladder inflammation may lead to diverse intraoperative difficulties. Severe local inflammation increases the surgical difficulty level. A good indicator of the severity of gallbladder inflammation should reflect the surgical difficulty level in a timely and objective manner. This might assist surgeons in making the decision to either convert to an open operation sooner or call for more experienced surgeons, as required [2]. Traditional indicators of surgical difficulty, such as length of hospital stay, operation time, blood loss, conversion rate, and morbidity rate, are unsuitable for use in intraoperative studies. These variables are easily influenced by the surgeon’s experience and proficiency. For example, an unskilled surgeon may require more time to complete LC for mild inflammation, resulting in more blood loss than a more proficient surgeon. However, it cannot be classified as a difficult surgical procedure in this scenario.

The Parkland Grading Scale (PGS) (Table 1) for cholecystitis has been reported to be a reliable and accurate predictor of LC outcomes [3]. It is a five-tiered, easy-to-implement grading system based on anatomical and inflammatory changes. In a previous study, [3] the levels of surgical difficulty correlating with different PGS grades were measured on a 5-point Likert-type scale ranging from 1 (least difficult) to 5 (most difficult). This is a subjective way to evaluate the difficulty level of a surgery.

**Table 1:** The Parkland Grading Scale for cholecystitis

Cholecystitis severity grade	Description of severity
**1**	Normal appearing gallbladder (“robin’s egg blue)
	No adhesions present
	Completely normal gallbladder
**2**	Minor adhesions at neck, otherwise normal gallbladder
	Adhesions restricted to the neck or lower of the gallbladder
**3**	Presence of any of the following:
	Hyperemia, pericholecystic fluid, adhesions to the body, or distended gallbladder
**4**	Presence of any of the following:
	Adhesions obscuring majority of gallbladder
	Grade 1–3 with abnormal liver anatomy, intrahepatic gallbladder, or impacted stone (Mirrizi)
**5**	Presence of any of the following:
	Perforation, necrosis, or inability to visualize the gallbladder due to adhesions

The difficulty score for intraoperative findings, established in the 2018 Tokyo Guidelines, is a novel surgical difficulty grading system (Table 2) [4, 5]. A Delphi consensus was reached among expert LC surgeons regarding the impact of intraoperative findings on surgical difficulty. A list of 25 key intraoperative findings that could potentially contribute to the surgical difficulty during LC was generated using the nominal group technique [6]. In this study, we used this novel grading system to ascertain whether the PGS system could accurately reflect the level of surgical difficulty.

**Table 2:** Surgical difficulty grading system for laparoscopic cholecystectomy

		Surgical Difficulty Score						
		0	1	2	3	4	5	6
Fibrosis/scarring of GB^a^								
	**Around the GB**	No findings		Fibrotic adhesion or partial scarring		Diffuse scarring		
	**Calot’s triangle area**	No findings		Sparse fibrosis	Dense fibrosis	Partial scarring	Diffuse scarring	
	**GB bed**	No findings	Sparse fibrosis	Dense fibrosis	Partial scarring	Diffuse scarring		
	**Additional findings of the GB and its surroundings**	No findings	Edematous change		Easy bleeding	Necrotic changes	Cholecystoenteric fistula	Cholecystocholedochal fistula (Mirizzi syndrome)
					Perforated GB wall and/or abscess formation	Abscess formation towards the liver parenchyma	Impacted gallstone in the confluence (Mirizzi syndrome)	
**Intra-abdominal factors unrelated to inflammation**		No findings	Non-inflammatory adhesion	Excessive visceral fat	GB neck mounting on the common bile duct	Inversion of the GB or collateral vein formation due to liver cirrhosis		
					Anomalous bile duct			

^**a**^ **Gallbladder**

## Methods

### Patient selection

The study protocol was approved by the Ethics Committee of Beijing Jishuitan Hospital. This retrospective study was conducted between January 2018 and February 2022. The data of a total of 261 patients who were diagnosed with cholelithiasis and cholecystitis and subsequently underwent LC were assessed. All treatment procedures were performed after informed consent was obtained from the patients. LC was performed by two experienced surgeons who each had performed this surgery for more than 800 patients. The PGS score and the surgical difficulty grading system were used to evaluate every surgical procedure by reviewing operative videos. Based on a previous study, [4] we defined the surgical difficulty level of each case by the highest value of the surgical difficulty scores listed in Table 2. Surgical difficulty scores ranged from 0 to 6. Traditional postoperative characteristics that were recorded included length of postoperative hospital stay, operation time, blood loss, conversion rate, and morbidity rate. A morbidity scoring system was used to analyze the severity of complications [7].

### Statistical analysis

Data processing and statistical analyses were conducted using SPSS 22.0 statistical analysis package. The Jonckheere-Terpstra test was performed for ordinal categorical variables to analyze the differences between the five PGS grades in terms of surgical difficulty scores. Spearman’s rank correlation analysis was performed to analyze the relationship between the PGS grades and surgical difficulty scores. The Mantel-Haenszel test was performed to evaluate the linear trend between morbidity scores and PGS grades. Moreover, *p*-values of < 0.05 were considered statistically significant.

## Results

The clinical characteristics of the patients are summarized in Table 3. This study included 109 men and 152 women with a mean age of 52.3 ± 14.44 years and mean body mass index of 24.83 ± 3.91 kg/m^2^. All 261 patients underwent LC. One patient required conversion to an open procedure due to Mirizzi syndrome. One patient developed choledocholithiasis 1 month after LC and subsequently underwent laparoscopic common bile duct exploration. No bile duct injury occurred during the operation in any of the cases. The mean operative time was 48 ± 24.85 min and mean length of postoperative hospital stay was 2.75 ± 2.77 days. The Parkland Grade was 1 for 56 patients, 2 for 57, 3 for 86, 4 for 34, and 5 for 28. Of the 261 patients, 13 developed complications after LC. The morbidity scoring system demonstrated the occurrence and severity of clinically relevant complications. The number of patients with relevant morbidities is displayed in Table 4.

**Table 3:** Baseline characteristics

	N (%)
Sex	
**male**	109 (41.8)
**female**	152 (58.2)
**Mean age, years (range)**	52.30 ± 14.44 (24–79)
**BMI** ^**a**^	24.83 ± 3.91
**CCI** ^**b**^	
**0**	192 (73.6)
**1**	59 (22.6)
**2**	9 (3.4)
**≥3**	1 (0.4)
**ASA-PS** ^**c**^	
**1**	136 (52.1)
**2**	104 (39.8)
**≥3**	21 (8.0)
**Conversion rate**	1 (0.4)
**Morbidity rate**	13 (5.0)
**PGS** ^**d**^	
**1**	56 (21.5)
**2**	57 (21.8)
**3**	86 (33.0)
**4**	34 (13.0)
**5**	28 (10.7)
**Surgical difficulty grading system**	
**0**	1 (0.4)
**1**	56 (21.5)
**2**	117 (44.8)
**3**	30 (11.5)
**4**	53 (20.3)
**5**	3 (1.1)
**6**	1 (0.4)
	**Mean ± SD** ^**e**^
**Length of stay post-operation, days**	2.75 ± 2.77
**Length of operation, minutes**	48.00 ± 24.85
**Blood loss, ml**	25.79 ± 18.59

^**a**^ **Body mass index;** ^**b**^ **Charlson comorbidity index;** ^**c**^ **American Society of Anesthesiologists physical status;** ^**d**^ **Parkland Grading Scale;** ^**e**^ **Standard deviation**

**Table 4:** Morbidity rates for morbidity score items

Complications	Score Points	N (%)
**Persistent abdominal pain**	1	3 (1.1)
**Persistent fever**	1	1 (0.3)
**Persistently raised signs of infection**	1	0
**Wound-healing complication**	2	2 (0.8)
**Thrombosis**	3	0
**Bleeding**	3	1 (0.3)
**Cholangitis**	3	2 (0.8)
**Icterus**	3	0
**Bile leakage**	3	1 (0.3)
**Abscess**	3	1 (0.3)
**Pneumonia**	3	0
**Embolic lung disease**	4	0
**Peritonitis**	4	0
**Pancreatitis**	4	1 (0.3)
**Renal failure**	4	0
**Relaparotomy**	5	1 (0.3)
**Cerebral ischemia or bleeding**	5	0
**Myocardial infarction**	5	0
**Septic shock**	5	0
**Death**	63	0

The Jonckheere-Terpstra test (trend test) demonstrated a significant difference between the five PGS grades in terms of surgical difficulty scores (*p* < 0.001, Table 5). In further pairwise comparisons, each grade (1–5) was significantly different from the others (*p* < 0.05) in terms of the difficulty level of surgery, except Grade 2 vs. 3 (*p* = 0.07) and Grade 3 vs. 4 (*p* = 0.08) (Table 6). Spearman’s rank correlation analysis demonstrated a significant correlation between the PGS grades and surgical difficulty scores (*r*_*s*_ = 0.681, *p* < 0.001). With an increase in the PGS level, the difficulty of the operation gradually increased.

**Table 5:** Jonckheere-Terpstra test for Parkland Grading Scale grades with respect to surgical difficulty scores

PGS^a^	N	Surgical difficulty score									Z-value	*p*-value
		0	1	2	3	4	5	6	Median	Mean rank		
**Grade 1**	56	1	45	7	3	0	0	0	1	48.38	20633.00	< 0.01
**Grade 2**	57	0	2	48	5	2	0	0	2	123.45		
**Grade 3**	86	0	9	44	12	21	0	0	2	145.28		
**Grade 4**	34	0	0	14	7	11	1	1	3	176.81		
**Grade 5**	28	0	0	4	3	19	2	0	4	212.13		

^**a**^ **Parkland Grading Scale**

**Table 6:** Pairwise comparisons of surgical difficulty scores of the five grades in the Parkland Grading Scale

PGS^a^ Pairwise Comparisons	Z-value	*p*-value
**1 vs. 2**	2807.50	< 0.01
**1 vs. 3**	4219.50	< 0.01
**1 vs. 4**	1802.50	< 0.01
**1 vs. 5**	1537.50	< 0.01
**2 vs. 3**	2959.00	0.070
**2 vs. 4**	1461.50	< 0.01
**2 vs. 5**	1439.50	< 0.01
**3 vs. 4**	1849.50	0.076
**3 vs. 5**	1907.50	< 0.01
**4 vs. 5**	649.00	0.041

^**a**^ **Parkland Grading Scale**

The Mantel-Haenszel test was performed to evaluate the linear trend between morbidity scores and PGS grades. Table 7 displays a significant linear association between them (*p* < 0.001). Spearman’s R value was 0.176 (*p* = 0.004). A linear trend was also tested between morbidity scores and surgical difficulty scores. It also demonstrated a distinct linear trend (*p* < 0.001, Table 8); Spearman’s R value was 0.178, (*p* = 0.004). This suggested that higher PGS grades and surgical difficulty scores were associated with higher morbidity scores.

**Table 7:** Mantel-Haenszel test for a linear trend between the Parkland Grading Scale and morbidity scores

Morbidity Score	N	PGS^a^					x2	*p*-value
		**1**	2	**3**	**4**	**5**		
**0**	248	56	55	82	31	24	10.0937.467	0.006
**1**	3	0	0	1	2	0		
**2**	2	0	0	1	1	0		
**3**	6	0	2	1	0	3		
**4**	1	0	0	0	0	1		
**5**	1	0	0	1	0	0		

^**a**^ **Parkland Grading Scale**

**Table 8:** Mantel-Haenszel test for a linear trend between surgical difficulty and morbidity scores

Morbidity Score	N	Surgical Difficulty Score							x2	*p*-value
		0	1	2	3	4	5	6		
**0**	248	1	56	112	29	47	3	0	10.093	0.001
**1**	3	0	0	2	0	1	0	0		
**2**	2	0	0	1	0	0	0	1		
**3**	6	0	0	2	0	4	0	0		
**4**	1	0	0	0	0	1	0	0		
**5**	1	0	0	0	1	0	0	0		

## Discussion

In recent years, there has been an increased focus on predicting the difficulty of LC procedures [8–11]. However, it is difficult to establish the definition of a difficult LC because the degree of difficulty depends on both patient-related factors and the surgeon’s surgical experience and skills [8]. Patient-related factors mainly include gallbladder inflammation. The PGS is a concise and efficient scale used for the real-time evaluation of the degree of gallbladder inflammation. If it could also objectively reflect the difficulty level of LC, it would have a significant impact on surgeon’s decision making, such as the decision to convert to a laparotomy procedure or ask for the assistance of expert LC surgeons. These decisions directly impact patient prognosis. Therefore, reliable predictions could effectively reduce the occurrence of postoperative complications. Although it is generally believed that a gallbladder with severe inflammation is more difficult to resect, an objective evaluation system to confirm this is still lacking.

The surgical difficulty scoring system established in the Japanese 2018 Tokyo guidelines is a detailed, scientific, and objective method to evaluate surgical difficulty. In a previous study, multiple evaluators assessed surgical difficulty items based on unedited videos and constructed the proposed surgical difficulty grading system [4] This provides a good basis for the evaluation of procedures in surgical research [12]. However, as it is a scoring system with up to 25 items, the scoring must be completed gradually during the surgical procedure or during the review of the surgical video. This process is time consuming, and the evaluation continues throughout the operation.

In comparison, PGS scoring can be performed at the beginning of the operation by observing the condition of the gallbladder and its surroundings after the laparoscope enters the abdominal cavity. Therefore, it is more efficient and less time consuming. Hence, if it is determined that the PGS is consistent in predicting surgical difficulty as a scoring system, the advantages of the simplicity and efficiency associated with the PGS will make it particularly useful to surgeons as a scoring system.

In this study, the Jonckheere-Terpstra test (trend test) demonstrated a significant difference in the surgical difficulty scores of the five levels of the PGS. There was a linear trend between the two scale systems. Spearman’s rank correlation analysis also demonstrated a strong correlation between the two variables. This illustrates that the PGS can accurately reflect the difficulty level of surgery objectively. However, pairwise comparison demonstrated no significant difference in surgical difficulty scores between PSG Grades 2 vs. 3 and Grades 3 vs. 4. This could be attributed to the failure of the PGS to estimate the inflammatory state of Calot’s triangle. The adhesion and scar status of Calot’s triangle have the greatest impact on the difficulty level of LC, and the surgical injury of this region plays a decisive role in the occurrence of intraoperative and postoperative complications (bleeding or bile leakage) [4]. There are four items related to fibrosis or scarring of Calot’s triangle included in the surgical difficulty grading system that the PGS does not include. In addition, in some cases, although peripheral adhesions cover the entire gallbladder, they are not dense and are easy to separate. The structure of Calot’s triangle can still be clearly dissected, and the operation is not excessively challenging. However, these cases are rated as Grade 5 on the PGS. This may account for the inconsistency observed between the PGS and surgical difficulty scores.

Both the PGS and surgical difficulty scores demonstrated good results in the trend test for morbidity scores. In our study, complications were scored to assess their severity. Classification of cases based solely on the presence or absence of postoperative complications does not accurately reflect the severity of morbidity. For example, puncture infection and bile leakage are both postoperative complications, but the prognoses of the two morbidities are very different. The Mantel-Haenszel test results demonstrated that the severity of morbidity increased with an increase in the PGS score. However, the low incidence of complications in this study may have biased the results and requires further research.

## Conclusions

We applied a novel surgical difficulty grading system to verify that the PGS can be used to precisely assess the surgical difficulty level of LC. The efficiency and conciseness of the PGS make it suitable for use in future research and clinical practice.

## Data Availability

The datasets analyzed during the current study are available from the corresponding author on reasonable request.

## Abbreviations

LC: Laparoscopic cholecystectomy
PGS: Parkland Grading Scale
